# Perception of stroke and knowledge of potential risk factors among Omani patients at increased risk for stroke

**DOI:** 10.1186/1471-2377-6-38

**Published:** 2006-10-20

**Authors:** Mohammed A Al Shafaee, Shyam S Ganguly, Abdullah R Al Asmi

**Affiliations:** 1 Department of Family Medicine and Public Health, College of Medicine and Health Sciences, Sultan Qaboos University, Muscat, Sultanate of Oman; 2 Department of Medicine, Neurology Unit, Sultan Qaboos University Hospital, Muscat, Sultanate of Oman

## Abstract

**Background:**

Previous studies have demonstrated poor knowledge of stroke among patients with established risk factors. This study aims to assess the baseline knowledge, among patients with increased risk for stroke in Oman, of warning symptoms of stroke, impending risk factors, treatment, and sources of information.

**Methods:**

In April 2005, trained family practice residents at Sultan Qaboos University Hospital Clinics (cardiology, neurology, diabetic, and lipid clinics), using a standardised, structured, pre-tested questionnaire, conducted a survey of 400 Omani patients. These patients all demonstrated potential risk factors for stroke.

**Results:**

Only 35% of the subjects stated that the brain is the organ affected by a stroke, 68% correctly identified at least one symptom/sign of a stroke, and 43% correctly identified at least one stroke risk factor. The majority (62%) did not believe they were at increased risk for stroke, and 98% had not been advised by their attending physician that their clinical conditions were risk factors for stroke. In the multivariable logistic regression analysis, lower age and higher levels of education were associated with better knowledge regarding the organ involved in stroke, stroke symptoms, and risk factors.

**Conclusion:**

Because their knowledge about stroke risk factors was poor, the subjects in this study were largely unaware of their increased risk for stroke. Intensive health education is needed to improve awareness of stroke, especially among the most vulnerable groups.

## Background

Stroke continues to be a leading cause of death and a major cause of long-term disability in adults worldwide. Previous studies have shown a poor level of knowledge of stroke among patients with established risk factors for stroke and in the community at large. Pancioli et al. [1], in a population-based survey, found that 57% of respondents identified at least 1 out of 5 known warning signs of stroke, and 68% correctly listed at least 1 risk factor. They also reported their study population mentioning various risk factors for stroke but being largely unaware of their own increased risk.

Carroll et al. [2] reported that patients with hypertension and those with history of smoking were significantly more likely to identify these as risk factors for stroke. Surprisingly, none of the patients with current stroke, and only one-third of the at-risk group who had suffered a previous stroke or transient ischaemic attack (TIA), recognised this as being a risk factor for further stroke.

Most patients and many caregivers did not recognise the onset of stroke and their knowledge of risk factors was poor [3]. Stroke knowledge was poorest among groups that had the highest risk of stroke [1,4,5].

Increasing the speed of presentation to hospital after the onset of stroke depends on the level of knowledge of stroke [6]. As a result, patients with stroke may fail to gain the benefits of acute treatments such as acute thrombolysis, because of the narrow therapeutic window [7]. Moreover, knowledge about the risk factors for stroke can help to prevent stroke in the first place [1]. Knowledge of stroke and awareness of its associated risk factors may improve adherence to medical advice regarding lifestyle modification. Systematic reviews have shown that one-time advice from healthcare workers during routine patient interactions can have an appreciable impact on patient behaviour [8,9]. However, persons at risk often tend to misunderstand their own risk, underestimating their probability for stroke and assuming that adverse events will not happen to them [10]. Samsa et al. [11] reported that about one-fourth of patients in their study who recalled being informed of their increased stroke risk by a physician nevertheless did not perceive themselves to be at increased risk for stroke.

The abovementioned studies point to the need for intensive public education about stroke, particularly in at-risk populations.

Oman, as a developing county, is undergoing several transitions. Lifestyles are changing rapidly, including changing dietary patterns (i.e., increasingly high-fat, high-salt and calorie-dense diets) and decreased physical activity. Non-communicable diseases have emerged as the dominant form of ill health in the country. This includes increasing prevalence of important risk factors for stroke (diabetes, hypertension and cardiovascular diseases).

To our knowledge there are no studies from Oman or other developing countries regarding knowledge of stroke among patients with established risk factors for stroke. The present study aims to assess baseline knowledge of warning symptoms of stroke, impending risk factors, treatment, availability of sources of information and the perceived risk of stroke among Omani patients with increased risk for stroke.

## Methods

### Sample and Setting

A random sample of 624 Omani patients who attended medical clinics (cardiology, neurology, diabetic, and lipid) at Sultan Qaboos University Hospital, a tertiary level referral hospital which receives patients from different parts of Oman, was selected from the appointment lists for the month of April 2005. We identified eligible subjects on the basis of diagnoses that placed them at increased risk for stroke. Any patient with one or more established risk factors for stroke (diabetes, hypertension, heart disease, dyslipidaemia, atrial fibrillation, smoking, previous stroke, transient ischaemic attack and carotid stenosis) was eligible to participate in the study.

### Procedure

Subjects were interviewed by 3 trained family practice residents. Each interviewer conducted a standardised, structured, one-to-one interview, in the local Arabic dialect, with open-ended questions. The interviewer assisted the interviewee only by clarifying any lack of understanding, if required. No attempts were made to prompt the subjects by suggesting answers directly.

### Measurement

A literature review was undertaken to uncover relevant items for the questionnaire [1,5,12]. Initially, a draft questionnaire was developed that addressed the awareness level of stroke warning signs and symptoms, risk factors, treatments, and sources of information. The questionnaire was pre-tested using a sample of 30 patients with established risk factors for stroke. The final questionnaire was divided into 2 sections. The first section gathered demographic information including age, sex, educational status, and diagnosis. The second section covered awareness of stroke in terms of etiology, organ involved, warning signs and symptoms, potential risk factors, information sources, perceived risk for stroke, treatment, and reaction to stroke symptoms.

This study was approved by the Research and Ethics Committee, College of Medicine and Health Sciences, Sultan Qaboos University, Sultanate of Oman.

### Statistical Analysis

The statistical analysis was carried out based on the description, distribution, and categorisation of variables included in the study. The association between study variables (i.e., components of stroke-related knowledge such as brain involvement, symptoms, and risk factors for stroke) and patient variables (age, gender, education level and diagnosis) was determined by an estimate of the difference in the proportions, using the chi-square test of significance. The relationship between the patient variables and knowledge about brain involvement, warning symptoms and signs, and risk factors were also evaluated in terms of odds ratios with 95% confidence intervals using separate logistic regression analyses. In each case, the knowledge-related variable was categorised as either 'aware' or 'unaware'. Wald statistics provided the statistical significance of the predictor variables. Missing data were excluded. Two-tailed significance tests were used, and a probability value of less than 0.05 was considered statistically significant. All data variables were processed and analysis was performed using SPSS version 10 for Windows and Epi Info™ version 6.

## Results

A total of 624 patients were randomly selected for the study and of those, 554 were eligible. The remainder of the patients were ineligible due to different diagnosis such as epilepsy and congenital heart diseases. A total of 400 patients agreed to participate in the study, a response rate of 72%. There were 210 (52.5%) men and 190 (47.5%) women. The mean age was 57 years with standard deviation (SD) of 9.5. The diagnosis, age and gender distributions of the enrolled subjects were not significantly different from that of the eligible population (mean age of 55 years and 1:1 male-to-female ratio). The details of the demographic and medical profiles of the subjects are shown in Table 1.

When asked about stroke causality, only 99 (24.8%) correctly stated that it is due to occlusion of a blood vessel or a haemorrhage in the brain. The majority, 225 (56.2%), gave incorrect answers and 76 (19.0%) were not sure. Of the subjects, 139 (34.8%) identified the brain as the organ of the body where a stroke occurs. However, the highest proportion (228, 57.0%) could not name the organ that is affected in stroke and 33 (8.3%) thought that stroke involves other organs in the body, particularly the nerves. In the multivariate logistic regression analysis, which assesses the effect of each patient characteristic after controlling for the effect of all other characteristics, younger age (*P *< 0.0001; OR 3.05; 95% CI, 1.89 to 4.92), male gender (*P *< 0.007; OR 1.91; 95% CI, 1.20 to 3.03), and higher level of education (*P *< 0.0001; OR 7.42; 95% CI, 3.47 to 15.89) were found to be significant predictors of a higher knowledge about the organ involved in stroke (Table 2).

### Knowledge of stroke symptoms

Table 3 shows the symptoms of stroke mentioned by the study subjects. The most common symptom identified by the majority of subjects (65.0%) was weakness and paralysis of one side of the body.

Of the subjects, 32.0% did not know a single warning symptom of stroke, 68.0% correctly mentioned at least 1 stroke symptom, 42.7% correctly mentioned at least 2 symptoms, and 26.0% correctly mentioned 3 or more symptoms. In the multivariate logistic regression analysis, younger age (*P *= 0.002; OR 2.26; 95% CI, 1.35 to 3.81) and higher level of education (*P *= 0.002; OR 6.91; 95% CI, 2.07 to 22.99) were found to be statistically significant predictors of a higher knowledge about stroke warning symptoms (Table 2).

### Risk factors for stroke

The most common risk factors for stroke mentioned by the subjects were hypertension (34.5%), diabetes (22.8%), cardiovascular diseases (10.8%), and hyperlipidaemia (8.5%) (Table 3).

Of the subjects, 44.3% could not mention a single risk factor and 12.7% mentioned false beliefs, such as cold weather and bad spirits, as risk factors for stroke. Only 43.0% correctly mentioned at least 1 risk factor, 28.5% correctly mentioned at least 2 risk factors, and 14.0% correctly mentioned 3 or more risk factors. When asked if they thought that they were at increased risk for stroke, a majority of subjects (62.0%) did not think that they were at increased risk, 30.8% did not know if they were at increased risk, and only a very small proportion (7.2%) thought that they were at increased risk for stroke. In the multivariate logistic regression analysis, younger age (*P *= 0.013; OR 1.81; 95% CI, 1.13 to 2.89) and higher level of education (*P *< 0.0001; OR 19.23; 95% CI, 4.58 to 80.81) were found to be statistically significant predictors of a higher knowledge about stroke risk factors (Table 2).

### Sources of information about stroke

The majority of subjects (95.5%) who have some information about stroke acquired such information through general life experiences and personal acquaintances (community 45.2%, relatives 39.3%, friends 14.7%, newspapers 3.7%, and television 0.8%). Surprisingly, only 4.5% of subjects had received information about stroke from health professionals (doctors 2.3%, and others 2.2%).

### Reaction to stroke symptoms

A majority (292 or 73.0%) of subjects reported that they would go immediately to a hospital emergency department if they recognised that they were having a stroke. Seventy-eight (19.5%) responded that they would try local (indigenous) treatment first and, if this did not work, they would visit a hospital. Moreover, 30 (7.5%) said they would like to be treated at home only with indigenous treatment.

### Knowledge of stroke treatment

Regarding the subjects' knowledge of the treatment of acute stroke, only 106 (26.5%) mentioned medical treatment in hospital and 62 (15.5%) suggested both medical and indigenous treatments. A large number (101 or 25.2%) of subjects mentioned indigenous treatment as the appropriate therapy for acute stroke and 131 (32.8%) believed that there is no appropriate treatment available.

## Discussion

This hospital-based study was conducted among Omani patients with known risk factors for stroke (i.e., diabetes, hypertension, heart disease, dyslipidaemia, atrial fibrillation, smoking, previous stroke, and transient ischaemic attack). Our study showed that younger age and higher level of education were associated with better knowledge about stroke mechanisms, the organ involved, stroke symptoms, and risk factors. Male gender was found to be correlated with better knowledge about stroke mechanisms, the organ involved, and stroke risk factors. In this sample, men were at higher level of education than women (*p *< 0.001). In some Western studies it was found that knowledge about stroke varies positively with education but is lower in men than women [1,12].

It appears from this study that some patients could not define what a stroke was or identify the affected organ, but they were able to recognise some of its symptoms or risk factors. The study has shown that only 24.8% of participants understood how stroke occurs. Pandian et al. [13], in a hospital-based survey of relatives of patients without history of stroke in India, reported that 30.8% of the study population mentioned that stroke occurs due to occlusion of a blood vessel in the brain. Moreover, in a population-based survey in Ireland, Parahoo et al. [14] found that the majority of the respondents were aware of the stroke mechanism.

A minority of subjects in this study identified the brain as the organ where a stroke occurs. The study from India [13] showed similar findings. In contrast, in a large prospective questionnaire study of elderly patients with established risk factors for stroke in the UK, Gupta et al. [5] found that 86% of respondents correctly correlated stroke with brain damage.

The most common symptom of stroke mentioned by subjects in our study was weakness and paralysis of one side of the body (65.0%). Similarly, Pandian et al. [13] found that the most common symptom identified by respondents was weakness of one side (62.2%). Kothari et al. [15], in a study of patients with acute stroke, reported that the most commonly documented stroke warning sign was weakness. Other common symptoms mentioned by our participants were speech difficulties (30.0%), and walking difficulties (25.8%). These latter symptoms were less frequently recognised by respondents in other studies from India [13], Australia [12], and the United States [1].

Hypertension was recognised as the most common risk factor for stroke in this study. This finding is similar to observations from studies in India [13], Michigan (USA) [4], and Cincinnati, Ohio (USA) [1]. Moreover, the proportion of subjects who mentioned other risk factors, such as diabetes, ischaemic heart disease, and high cholesterol was higher compared with the community-based studies [12,13]. Although this study was conducted among patients with known risk factors for stroke, the majority of these patients (57%) could not name a single correct risk factor for stroke. Yoon et al. [12] and Pandian et al. [13] reported that the awareness of stroke risk factors among high-risk individuals was poor and did not differ significantly from those of respondents who had no risk factors. Gupta et al. [5] reported that 65% of the elderly patients in their study correctly identified at least one risk factor for stroke.

Our present study has shown that only a very small proportion (7.2%) believed that they were at increased risk of stroke as a result of their underlying medical conditions. Gupta et al. [5] found that 15% of patients considered themselves at increased risk of stroke because of underlying disease. Carroll et al. [2] reported that none of the stroke patients, and only a third of the at-risk group who had suffered a previous stroke or TIA, recognised this as being a risk factor for further stroke.

A majority of subjects in this study (73.0%) mentioned that they would go immediately to a hospital emergency department if they recognised that they were having a stroke. This result is similar to findings from other studies [4,12-14]. However, if they were asked how they would react to particular symptoms, without reference to stroke, they might have responded differently.

In this study, 19.5% of subjects mentioned that they would like to be treated at home first with indigenous treatments, but that if this did not work, they would visit a hospital. Moreover, 7.5% would like to be treated exclusively at home and only with indigenous treatments. There are strong beliefs in the Omani population regarding the treatment of stroke. Many prefer to isolate the patient in a warm place at home where only one person can take care of him/her, which is referred to as "Kanan" (complete isolation), because they believe that heat and isolation will speed recovery. "Wasam", or branding, might also be used by local healers on certain points on the patient's body. There are also treatments utilizing herbs and herbal oil massage.

One implication of our results is the importance of increasing public awareness about stroke and stroke prevention, particularly in the at-risk population. Because of the high illiteracy rate among people at risk in Oman, we recommend that health education messages be disseminated via audio-visual materials rather than text materials as is currently the case. We also recommend initiation of a comprehensive community education campaign consisting of media appearances by local stroke experts to address the issues of stroke, particularly stroke treatment, and to correct the misconceptions about issues involving stroke.

Our study has encountered several limitations. The interviewers were trained on how to avoid asking leading questions. Nonetheless, because of the low level of education of some subjects, it is possible that the interviewers might have influenced the participants while they were trying to explain the meaning of the questions to them. Therefore, our study is not immune from interviewer bias. This bias can be expected to lower the observed prevalence of unawareness among participants. Thus, if the effect of bias is removed, the observed prevalence would be expected to increase which further supports our finding of a high-level of unawareness. Another limitation is that a few participants (<2%) were given only an abbreviated interview in the clinics because of other commitments. This might have affected the validity of the answers they provided.

## Conclusion

This is the first study in Oman to assess stroke knowledge in patients at risk. Not surprisingly, we found that young patients with higher levels of education were more knowledgeable about stroke. However, the current Omani population at risk of stroke is elderly, most of which is illiterate. In addition, the majority of the study subjects have acquired stroke knowledge from life experience rather than from their doctors. Therefore, there is an urgent need to develop health education programmes to improve the awareness of stroke both at the primary and the secondary healthcare levels. These educational programmes should be tailored for the population at risk. In addition, further studies are needed in this area, particularly in the indigenous treatment of stroke in Oman, its benefits, and its potential complications. The findings of these studies may then provide further guidance to future health education programmes.

## Competing interests

The authors declare that they have no competing interests.

## Authors' contributions

MAAS designed the study, analysed the data and drafted the manuscript. SSG participated in the statistical analysis of the data and the interpretation of results. ARA interpreted the results and the general conclusions. All authors read and approved the final manuscript.

## Pre-publication history

The pre-publication history for this paper can be accessed here:



## Figures and Tables

**Table 1:** Demographic and Medical Profile of High Risk Patients (N = 400).

**Characteristics**	**No**	**(%)**
Age, y (mean ± SD)	57.0 ± 9.5	
41 – 50	125	31.3	
51 – 60	145	36.3
61 – 70	100	25.0
71 +	30	7.5
Sex		
Male	210	52.5
Female	190	47.5
Education		
Illiterate	169	42.2
Read & Write	117	29.2
Elementary & Preparatory	39	9.8
Secondary	27	6.8
College & University	48	12.0
Current health status (diagnosis)		
Cardiovascular disease	208	52.0
Hypertension	203	50.8
Diabetes mellitus	178	44.5
Dyslipidaemia	80	20.0
Previous stroke	25	6.3
Smoking	20	5.0
Atrial fibrillation	9	2.3
Transient ischaemic attack	4	1.0
Carotid stenosis	0	0.0

**Table 2:** Multivariate Analysis: Predictors of Knowledge about Brain Involvement, Warning Symptoms, and Risk Factors for Stroke.

**Variables**	**Brain Injury**		**Symptoms & signs**		**Risk factors**	
	*OR (95%CI)*	p-*value*	*OR (95%CI)*	p-*value*	*OR (95%CI)*	p-*value*
Age (years)		<0.0001		0.002		0.013
> 50	1.00		1.00		1.00	
≤ 50	3.05 (1.89, 4.92)		2.26 (1.35, 3.81)		1.81 (1.13, 2.89)	
Sex		0.007		0.647		0.158
Female	1.00		1.00		1.00	
Male	1.91 (1.20, 3.03)		1.11 (0.71, 1.72)		1.35 (0.89, 2.06)	
Education		<0.0001		0.002		<0.0001
Up to secondary	1.00		1.00		1.00	
College & above	7.42 (3.47, 15.89)		6.91 (2.07, 22.99)		19.23 (4.58, 80.81)	

**Table 3:** Participants' Knowledge of Stroke Warning Symptoms and Risk Factors (N = 400).

**Variables**	**No.**	**(%)**
**Symptoms & signs**		
Weakness/paralysis	260	65.0
Speech difficulty	120	30.0
Walking difficulty	103	25.8
Personality changes	30	7.5
Numbness	29	7.3
Dizziness	22	5.5
Headache	13	3.3
Visual difficulty	12	3.0
Swallowing difficulty	11	2.8
Bladder incontinence	4	1.0
		
**Risk factors/causes**		
Hypertension	138	34.5
Diabetes	91	22.8
False beliefs*	51	12.7
IHD (ischaemic heart disease)	43	10.8
Hyperlipidaemia	34	8.5
Obesity	23	5.8
Smoking	21	5.3
Excessive alcohol intake	16	4.0
No physical activity	13	3.3
Older age	8	2.0
Previous stroke	3	0.8
Transient ischaemic attack	3	0.8

*False beliefs refer to non-established risk factors for stoke such as weather (hot or cold) and bad spirits.
